# Estimation of energy flow and environmental impacts of quinoa cultivation through life cycle assessment methodology

**DOI:** 10.1007/s11356-020-08576-9

**Published:** 2020-04-12

**Authors:** Amin Lotfalian Dehkordi, Marziye Forootan

**Affiliations:** Department of Mechanical Engineering of Biosystems, Faculty of Agriculture, Shahrekord University, PO Box 115, Shahrekord, 88186-34141 Iran

**Keywords:** Agricultural machinery, Energy ratio, Environmental pollutants, Chemical fertilizers, Quinoa

## Abstract

Quinoa is an adaptable plant that is rich in terms of nutritional properties. Currently, the promotion and cultivation of quinoa are expanding in Iran. The present study aimed to investigate the energy consumption of quinoa grain production and its environmental impacts through life cycle assessment. In this regard, in order to evaluate the environmental and energy indices, required data were collected from quinoa farmers in Isfahan. The high energy ratio (ER > 1) and positive net energy show that quinoa cultivation is efficient. Based on the results, irrigation water and nitrate fertilizer were identified as the major contributors to energy consumption. Based on the normalization method, the highest and lowest environmental impacts during the production process were related to the indices of marine aquatic ecotoxicity and ozone layer depletion, respectively. Results showed that in the global warming potential impact, 354 kg CO_2eq._ were emitted per production of 1 tonne of quinoa grain. Diesel fuel and nitrogen fertilizer had a significant effect on most environmental impacts. Proper management of chemical fertilizers and agricultural machinery are key factors for sustainable cultivation of quinoa.

## Introduction

Agricultural crops play an important role in human life. Population growth requires increased food production and consequently increased the quantity and quality of agricultural products. Most regions of Iran are located in arid and semiarid areas with high salinity soil. Quinoa (*Chenopodium quinoa*) has considerable resistance to a wide range of abiotic stresses such as cold, salinity, and water stress (Jacobsen et al. 2009). Quinoa is cultivated in most parts of the world due to the high quality of quinoa grain and its high production potential in tough cultivation conditions (Gomez-Pando and Eguiluz-de 2013). This crop can be used directly as human food (after Saponin removal) or can be processed (bread, cake, and pasta). Since quinoa is rich in terms of protein, it is turning to be a suitable alternative for rice. The protein content of quinoa is two times higher than it in wheat, and it is one of the few non-meat proteins that is qualitatively and quantitatively better than other plant proteins (Ceccato et al. 2011).

According to the latest FAO statistics, in the worldwide, quinoa grain have been harvested as 146,735 tonnes from 173,242 ha farming area with an average crop yield of 847 kg ha^−1^ (FAO 2017). Dependence on energy resources in developed and developing countries has led to serious environmental, technical, commercial, and even social problems (Safa and Samarasinghe 2011). Efficient use of energy causes increased production, productivity, economy, profitability, and sustainable competition in agriculture. Energy saving is important for sustainable development in agricultural systems (Pishgar-Komleh et al. 2012). In order to use energy in an efficient way, it is necessary to know the pattern of energy consumption (Heidari et al. 2011). Given the high number of factors affecting agricultural production, it is difficult to calculate the energy input in agricultural production compared with the industrial sector. Therefore, the energy consumption pattern and its efficiency in the agricultural system were investigated and analyzed in this study. One of the prerequisites for achieving the sustainable agriculture is paying attention to environmental protection, identifying and reducing environmental pollutants. Life cycle assessment (LCA) is a tool to measure the environmental consequences of a product during its production cycle, or in other words, from the beginning to the end of the product formation process (cradle to grave approach). Also, the energy efficiency of a product can be estimated by LCA methodology. In this method, all the required steps and inputs used in the process are investigated. Despite the importance of the quinoa cultivation in Iran and considerable amount of research works that focused on the assessment of the energy use and environmental impacts of agricultural products, there is no serious attempt on these topics in the cultivation of quinoa. Only one study occurred on the environmental impacts of quinoa grain in Peru. Their results indicated that the environmental impacts of quinoa production were in the range of other agricultural products, but quinoa has less pollution than other protein products, especially animal protein (Cancino-Espinoza et al. 2018). In a comparative LCA study of winter wheat and summer maize in China, the results showed that the impacts of abiotic depletion and eutrophication in both of the systems contributed the most to the environmental pollutions (Wang et al. 2007). Pellizzi (1992) studied the energy balance of corn production. Based on their results, the specific energy value was obtained as 4.2–8.4 MJ kg^−1^. The total energy consumption of the total production area was calculated to be 13 Mt of oil equivalent per year. Safa et al. (2009) reported that total input energy for irrigated and rainfed wheat production was 25,600 and 17,450 GJ/ha^−1^, respectively, in New Zealand. In that research, chemical fertilizer and electricity played the most important role in irrigated farms output with 10,190 and 3430 GJ/ha^−1^, respectively. Nemecek et al. (2011) compared the environmental burdens of organic farming vs. integrated production systems in Switzerland. They showed that the N_2_O and CO_2_ emissions from chemical fertilizers made high contributions to GWP. In Chile, Iriarte et al. (2010) concluded that the N-based fertilizers had significant effects on the five impact categories of acidification potential, eutrophication, global warming potential, human toxicity potential, and marine aquatic ecotoxicity in sunflower and rapeseed productions.

The higher efficient use of energy and better environmental management in quinoa cultivation are important to provide a sustainable and cost-effective production, and was recognized as an important tool for both farmers and decision-makers in agriculture. To the best of authors’ knowledge, there is no study up to date on the evaluation of energy consumption and environmental impacts in the quinoa production using the LCA approach in Iran.

For the first time in 2018, the quinoa was cultivated in Isfahan province of Iran to meet the food demands of those poorer populations. Accordingly, our objectives of this research were (1) to examine the quantity of energy used for quinoa production in the Isfahan province, Iran, and (2) to estimate the environmental impacts emitted from 1 tonne of quinoa production by LCA.

## Materials and methods

### Sampling and data collection

The present study was conducted in Isfahan province which is located in the center of Iran (30° 43′–34° 27′ N/49° 36′–55° 31′ E) with the area of about 105,937 km^2^. This region has different climatic zones as well as different soil texture and salinity (Anonymous 2018). In order to assess the life cycle of the quinoa, required data relate to crop cultivation was collected from 30 quinoa growers in Isfahan province of Iran. Questionnaires and face-to-face interviews with growers were applied to analyze the energy consumption and environmental impacts. Therefore, due to limitation of population size, there is no need to the sampling and population size is equal to sample size. The completed inventory of inputs includes machinery used for agricultural operations, fuel consumption, chemical fertilizers, manure, insecticides, seed, human labor, water for irrigation, and electricity as well as outputs including quinoa grain and crop residues. The energy equivalent of inputs used in quinoa cultivation was selected from previous literature. Energy equivalent of quinoa grain and residues were measured in the laboratory using a calorimeter bomb (Table 1).

**Table 1:** Consumed and produced energy for the production of quinoa in one hectare

Input–output (unit)	Energy equivalent (MJ unit^−1^)—references	Average quantity (unit ha^−1^)	Consumption energy (MJ ha^−1^)	Share of input energy (%)
A. Inputs				
1. Seed (kg)	17.21—calculated	8.39	144.56	0.56
2. Chemical fertilizer (kg)				
2.1. Nitrate (N)	78.1—Rajaeifar et al. 2013	82.57	6449.1	25.27
2.2. Phosphate (P_2_O_5_)	17.4—Rajaeifar et al. 2013	42.25	735.15	2.88
2.3. Potassium (K_2_O)	13.7—Rajaeifar et al. 2013	36.24	496.62	1.94
2.4. Sulfur	1.12—Rajaeifar et al. 2013	25.50	28.57	0.11
3. Manure (kg)	0.3—Elhami et al. 2016	3535.70	1060.71	4.15
4. Machinery (h)				
4.1. Tractor	93.61—Rafiee et al. 2010	6.73	630.30	2.47
4.2. Machinery	62.70—Rafiee et al. 2010	6.98	438.00	1.71
4.3. Combine	87.63—Rafiee et al. 2010	2.15	220.30	0.86
5. Insecticide (kg)	216—Pishgar-Komleh et al. 2012	1.25	126.50	0.49
6. Diesel fuel (L)	47.8—Pishgar-Komleh et al. 2012	117.83	5632.43	22.07
7. Labor (h)	1.96—Elhami et al. 2016	113.97	223.40	0.87
8. Electricity (kWh)	11.93—Elhami et al. 2016	228.57	2726.86	10.68
9. Water for irrigation (m^3^)	1.02—Pishgar-Komleh et a. 2012	6500	6630.00	25.98
Total inputs energy			25,513.93	100
B. Outputs (kg)				
1. Quinoa grain	17.21—calculated	1590.83	27,378.30	34
2. Crop residues	12.13—calculated	4236.10	51,384.00	66
Total outputs energy		5826.93	78,762.30	100

### Energy analyses

Improving the productivity of energy cycle in agriculture is one of the main measures to improve energy consumption, save money, conserve natural resources, and reduce environmental pollution (Pahlavan et al. 2012). In order to compare and evaluate the energy input and output of the system, standard indices including energy ratio (ER), energy productivity (EP), specific energy (SE), and net energy gain (NEG) are calculated using Eqs. 1, 2, 3, and 4, respectively (Naderi et al. 2019). Energy demand in agriculture can be classified into direct and indirect energies or renewable and non-renewable energies. In the present study, human labor, fuel, electricity, and irrigation water were considered as direct energy (DE) and all types of fertilizers, pesticides, seeds, and machinery were considered as indirect energy (IDE) resources. Renewable energy (RE) includes human labor, seeds, manure, and irrigation water, and non-renewable (NRE) resources include electricity, machinery, fuel, pesticides, and fertilizers (Erdal et al. 2007).

1 $$ \mathrm{Energy}\ \mathrm{use}\ \mathrm{efficiency}=\mathrm{output}\ \mathrm{energy}\ \left(\mathrm{MJ}\ {\mathrm{ha}}^{-1}\right)/\mathrm{input}\ \mathrm{energy}\ \left(\mathrm{MJ}\ {\mathrm{ha}}^{-1}\right) $$

2 $$ \mathrm{Energy}\ \mathrm{productivity}=\mathrm{quinoa}\ \mathrm{output}\ \left(\mathrm{kg}\ {\mathrm{ha}}^{-1}\right)/\mathrm{input}\ \mathrm{energy}\ \left(\mathrm{MJ}\ {\mathrm{ha}}^{-1}\right) $$

3 $$ \mathrm{Specific}\ \mathrm{energy}=\mathrm{input}\ \mathrm{energy}\ \left(\mathrm{MJ}\ {\mathrm{ha}}^{-1}\right)/\mathrm{quinoa}\ \mathrm{output}\ \left(\mathrm{kg}\ {\mathrm{ha}}^{-1}\right) $$

4 $$ \mathrm{Net}\ \mathrm{energy}\ \mathrm{Gain}=\mathrm{output}\ \mathrm{energy}\ \left(\mathrm{MJ}\ {\mathrm{ha}}^{-1}\right)-\mathrm{input}\ \mathrm{energy}\ \left(\mathrm{MJ}\ {\mathrm{ha}}^{-1}\right) $$

### Life cycle assessment

Life cycle assessment (LCA) methodology consists of four stages: goal and scope definition, life cycle inventory (LCI), life cycle impact assessment (LCIA), and interpretation of results (ISO14040).

#### Goal and scope

**Fig. 1:**
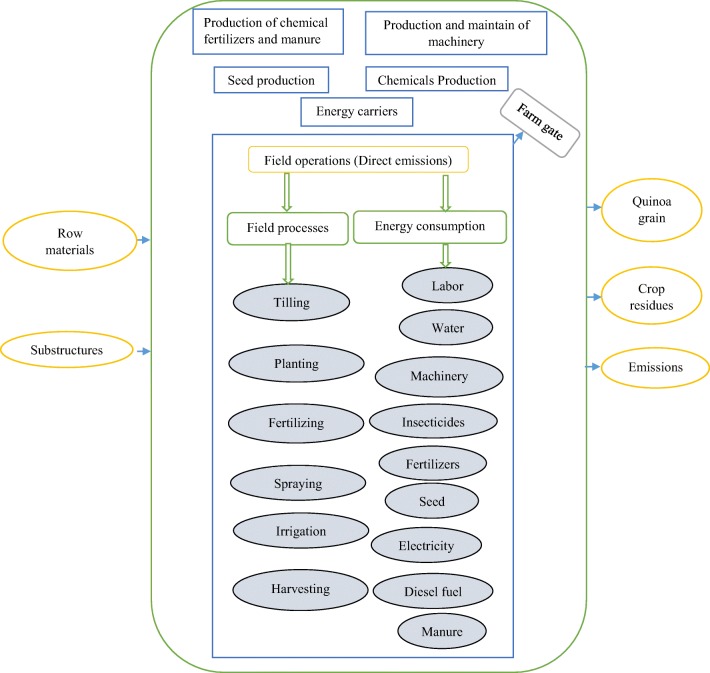
The farm gate as system boundary of quinoa production

The first stage of the LCA study is defining the goal and scope. The purpose of this study was to investigate the environmental impacts and energy consumption pattern for quinoa production. All operations involved in the life cycle, products, and processes must be specified. In the present study, system boundary was defined as farm gates, including all field operations such as tillage, planting to harvesting operations, all agricultural inputs (manure, chemical fertilizers, insecticides, and seeds), agricultural machinery, human resources, diesel fuel, water, and also output products including quinoa grain, crop residues, and emissions. It should be mentioned that the stages in this study are solely concerned with quinoa production and do not include the process of Saponin removal. Figure 1 indicates the system boundary. This study is a cradle to farm gate study in which the whole life cycle from inputs production to quinoa production were considered and investigated. The FU is the quantitative description of the production system that is used as a source in the LCA study (Sahle and Potting 2013). In this study, FUs were chosen: mass-based (1 tonne of quinoa grain) and land-based (1 ha of area under cultivation).

#### Life cycle inventory

Life cycle inventory (LCI) includes the measurement of various input materials, energy flows and environmental emissions. Data related to the foreground system (farm operations in quinoa production or inputs consumption) were directly collected using face-to-face questionnaire (third column of Table 1) and data from the background system (production of inputs) extracted from the Ecoinvent ®3.0 database available in Simapro V. 8.2.3 software, previous literatures, and IPCC guidelines that are shown in Table 2. Also, the equations of direct emissions are brought in the AppendixS1 (Nemecek et al. 2014; Nemecek and Kagi 2007; IPCC 2007; Pre-Consultants 2014; Wikström and Adolfsson 2004).

**Table 2:** Coefficients for calculating the direct emissions related to application of inputs in quinoa production

Direct emissions	Emission factors (kg/unit)
1. From chemical fertilizers and manure (kg)	
1.1. Dinitrogen monoxide to air	0.001 × [1]
1.2. Dinitrogen monoxide to air	0.01 × [1.557]
1.3. Carbon dioxide to air	0.2 × [3.666]
1.4. Ammonia to air	0.1 × [1.214]
1.5. Ammonia to air	0.2 × [1.214]
1.6. Nitrogen oxide to air	0.21 × [1]
1.7. Phosphorus to water	0.03 × [4.428]
1.8. Nitrate to water	0.05 × [0.436]
2. From diesel fuel to air (MJ)	
2.1. Carbon dioxide	7.45E−02
2.2. Sulfur dioxide	2.41E−05
2.3. Methane	3.08E−06
2.4. Dinitrogen monoxide	2.86E−06
2.5. Ammonia	4.77E−07
2.6. Hydrocarbons	7.85E−08
2.7. Nitrogen oxide	1.06E−03
2.8. Carbon monoxide	1.50E−04
2.9. Particulates (b2.5 μm)	1.07E−04
3. From residue burning to air (kg)	
3.1. Methane	5.00E−03
3.2. Dinitrogen monoxide	7.00E−03
3.3. Nitrogen oxides	1.21E−02
3.4. Carbon monoxide	6.00E−02
4. From human labor to air (man-h)	
4.1. Carbon dioxide	7.00E−01

In this study in order to calculate the direct emissions, we used following assumptions.

Based on the IPCC guideline (2007), 1.25 kg NO_x_ is emitted to the air per 100 kg N-based fertilizer (Galloway et al. 1995). The amount of ammonia released from urea fertilizer is about 17% of the total nitrogen of urea fertilizer (Goebes et al. 2003; Brentrup et al. 2000). The recorded amount of CO_2_ emitted from the urea fertilizer is 1570 g per 1 kg of urea in the Ecoinvent database. Also, 30% of the total N-based fertilizer emits to the groundwater in the form of nitrate (Erickson et al. 2001). The emissions from phosphate fertilizers were calculated based on Nemecek and Kagi (2007). The average amount of phosphorus emitted into groundwater is about 0.07 kg per 1 ha (Nemecek et al. 2014).

Different pesticides with different active ingredients are used in the study area. Thirty to 50% of all pesticides are emitted into the air. Spraying operations and evaporation are the most important factors in the emission of these substances (IPCC 2007). In the Ecoinvent database, 100% of pesticides are assumed to emit into agricultural soils (Nemecek and Kagi 2007). The only fuel used in the system is diesel fuel. Emission factors of diesel fuel were extracted from Ecoinvent database to calculate the total diesel fuel emissions. Based on that, direct emissions of diesel consumption were calculated by Nemecek and Kagi (2007) and emissions of 9 pollutants were quantified for diesel fuel.

#### Life cycle impact assessment and interpretation of results

In this stage, the environmental impacts of each input, output, and operation were determined in each of the environmental category. For this purpose, Simapro environmental software was used. Then the obtained results were analyzed and compared with those obtained from similar studies. Environmental impacts of quinoa production were identified and possible solutions were recommended to correct the environmental hotspots and enhance the positive impacts. In the software database, a lot of information about each particular product around the world was stored. The amount of all environmental inputs and emissions were calculated using the above coefficients and formulas and were entered into Simapro V.8.2.3 software, then they were analyzed using CML-IA baseline V3.01/EU25 modeling approach in the form of some environmental impact categories. According to ISO 14040 standard (2006), the stage of life cycle impact assessment consists of two steps: a mandatory step and an optional step. The mandatory step includes the selection of impact categories and categorization, and the second step includes normalization and weighting. In the normalization step, the values of each category of impact are divided into a reference value. Typically, this reference is the average annual environmental impact in a country or climate per person. However, since this amount has not been calculated for this purpose in Iran, the exact results cannot be obtained. On the other hand, the mandatory steps cover the objectives of this study. In such a way, the index can be calculated for each impact category, and, on the other hand, the inputs with a greater impact on each category are identified. Therefore, in this study, only the mandatory steps were performed. Accordingly, eleven impact categories including global warming potential (GWP), abiotic depletion (AD), fossil fuels abiotic depletion (FAD), eutrophication (EU), ozone layer depletion (OLD), human toxicity potential (HTP), terrestrial ecotoxicity (TE), freshwater aquatic ecotoxicity (FAE), marine aquatic ecotoxicity (MAE), photochemical oxidation (PhO), and acidification potential (ACP) were selected as the most important impact categories and were studied for the initial data analysis (Goedkoop et al. 2008).

## Results and discussion

### Pattern of energy consumption

The results of energy analysis of quinoa production are presented in the fourth row of Table 2. As can be seen, total inputs energy was calculated to be 25,514 MJ ha^−1^, from which water for irrigation, nitrate fertilizer, diesel fuel, and electricity were the main contributors and were consumed as 6630 MJ ha^−1^ (26%), 6449 MJ ha^−1^ (25.27%), 5632 MJ ha^−1^ (22.07%), and 2727 MJ ha^−1^ (10.68%), respectively. The results also revealed that the insecticide and seed had the least demanding energy inputs for quinoa production. The use of appropriate irrigation strategies such as drip irrigation can reduce water and electricity consumption. In the life cycle study of wheat, electricity, chemical fertilizer, and irrigation were the most influential inputs on energy consumption (Khoshnevisan et al. 2013). In a study that was conducted on potato energy flow in Isfahan province, the highest energy consumption was related to chemical fertilizers (Pishgar-Komleh et al. 2012). The difference between chemical fertilizer and organic fertilizer consumption indicates that increasing yield using more chemical fertilizer consumption cannot be an appropriate alternative. Diesel fuel is used for tractors and agricultural machinery. The shortage of complex machinery has increased the number and traffic of tractors in farms which causes high fuel consumption. Inefficient irrigation causes excessive water consumption and increases water loss. Also, output energy of quinoa grain and crop residues were found to be 27,378 MJ ha^−1^ and 51,384 MJ ha^−1^, respectively.

The results related to energy indices in quinoa cultivation are reported in Table 3. The ER without considering crop residues was calculated as 1.07 and the NEG was 1864 MJ ha^−1^, which indicates efficient quinoa grain production. As a result, the ER of quinoa is lower than the rapeseed oil. It can be attributed to lower energy consumption in quinoa compared with rapeseed (Mousavi-Avval et al. 2011; Choobin et al. 2016). Currently, the residue of cultivated quinoa is used as either livestock feed or fertilizer or can be burnt by farmers. The total ER for quinoa cultivation (quinoa grain and crop residues) was obtained as 3.08. This amount indicates the necessity of the residues retain managing in the quinoa cultivation. EP and SE indices were compared in two scenarios as considering just quinoa production as first scenario and quinoa grain production and its residues as second scenario and results showed that about two-thirds of the consumed energy in production is related to the crop residue.

**Table 3:** Calculated energy indices for quinoa production in Isfahan province, Iran

Indices	Units	Quantity of energy indices		Contribution of energy forms (%)
		Quinoa grain	Quinoa grain + crop residues	
Energy ratio	-	1.07	3.08	
Energy productivity	kg MJ^−1^	0.06	0.22	
Specific energy	MJ kg^−1^	16.66	4.54	
Net energy gain	MJ ha^−1^	1864.37	5324.37	
Direct energy	MJ ha^−1^			15,212.69 (68%)
Indirect energy	MJ ha^−1^			10,301.24 (32%)
Renewable energy	MJ ha^−1^			8058.67 (32%)
Non-renewable energy	MJ ha^−1^			17,455.26 (68%)

Higher accuracy in timing, amount, and type of fertilizers can be effective in increasing the grain to residue ratio. The difference in the amount of RE and NRE can be attributed to chemical fertilizers and fossil fuels. Consumption of NRE resources has hazards for environment. Sustainable agriculture and a healthier environment can be achieved by proper fertilizer management, applying advanced machinery, and renewable fuels. The amount of IDE is higher than DE that can be attributed to chemical fertilizers. This gap can be reduced through proper management of chemical fertilizers. Table 3 shows the distribution of total energy input as DE vs. IDE and RE vs. NRE. The results revealed the contribution of 68% and 32% of total energy input for DE and IDE, respectively. The shares of RE and NRE are 32% and 68% of total energy input. Several researches have shown that the contribution of DE is higher than that of IDE, and the share of NRE is more than that of RE in production of different agricultural products (Rafiee et al. 2010; Mohammadi and Omid 2010; Kizilaslan 2009).

### Life cycle impact assessment

**Fig. 2:**
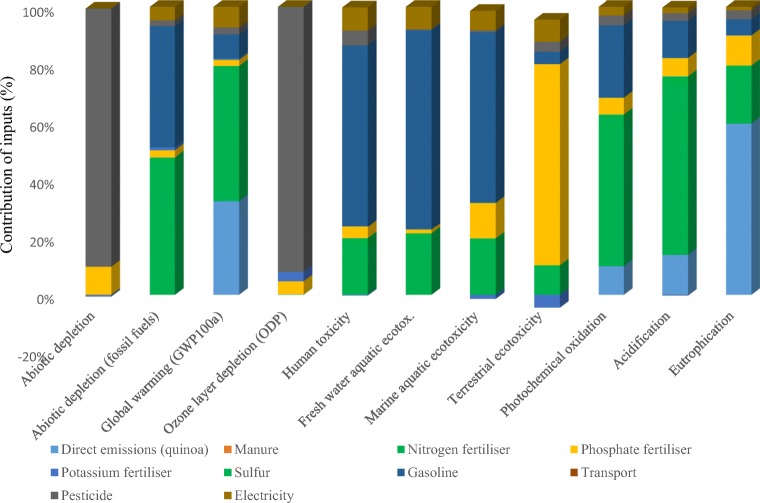
The role of inputs in the rate of environmental indices of quinoa production

Plants are both pollutants and environmental cleaners, simultaneously. Investigating the life cycle of plants can reduce pollutants. The values of environmental impact categories on the basis of the mass-based and land-based FUs in quinoa cultivation are presented in Table 4. The values of environmental impact categories related to 1 ha of quinoa cultivation were approximately 1.6 times the relevant impact categories for one tonne of produced quinoa. This can be attributed to the yield of quinoa grain which is 1.6 tons per ha. Also, to better understand the sources of environmental emissions, impact categories associated with direct (on-farm) and indirect (off-farm) emissions of quinoa cultivation are reported in Table 4. Potential for fossil resources depletion is determined based on available reserves and extraction rates. As can be seen in Table 4, FAD was obtained as 7292 MJ per 1 tonne of quinoa production. This is about half of this index for rapeseed (Choobin et al. 2016). Figure 2 shows the contribution of each input to total environmental impacts for each impact category. Diesel and nitrogen fertilizers are identified as hotspots for FAD impact.

**Table 4:** Values of the environmental impact in quinoa grain production per two distinctive FUs

Impact categories	Nomenclature	Units	Direct	Indirect	Total	
					Mass-based FU, 1 tonne	Land-based FU, 1 ha
Abiotic depletion	AD	g Sb_eq._	0	1.8	1.8	2.97
Abiotic depletion (fossil fuels)	FAD	MJ	0	7291.80	7291.80	12,031.47
Acidification potential	ACP	kg SO_2eq._	0.683	2.552	3.190	5.263
Eutrophication	EU	g PO_4_^−3^_eq._	272	223	495	817
Global warming potential	GWP	kg CO_2eq._	106.19	247.79	353.99	584.08
Ozone layer depletion	OLD	mg CFC11_eq._	0	19	19	31.35
Human toxicity	HTP	kg 1,4-DB_eq._	0	280.270	280.270	462.445
Fresh water aquatic ecotoxicity	FAE	kg 1,4-DB_eq._	0	92.021	92.021	151.834
Marine aquatic ecotoxicity	MAE	kg 1,4-DB_eq._	0	395,330.04	395,330.04	652,294.56
Terrestrial ecotoxicity	TE	g 1,4-DB_eq._	0	155	155	255.75
Photochemical oxidation	PhO	g C_2_H_4 eq._	16	145	161	265.65

GWP is the creation of global warming over a 100-year period that is generated by materials released during the production of products. In the present study, this impact was calculated as 354 kg CO_2eq._ for the production of one tonne of quinoa. The results of a study in Peru indicated that 880 kg CO_2eq._ is emitted per production of 1 tonne of packaged organic quinoa (Cancino-Espinoza et al. 2018). In a study by Habibi et al. (2019), the effect of rice cultivation on GWP impact was analyzed in Amol and Rasht counties and obtained as 277 and 276 kg CO_2eq._ per tonnes of rice, respectively. Figure 2 shows that the most effective factors on the GWP impact are nitrogen fertilizer and on-farm emissions (direct emissions) which is in agreement with results obtained from a study on canola cultivation in Isfahan province (Khanali et al. 2018). Elhami et al. (2016) found agricultural machinery and chemical fertilizers as the most important inputs affecting GWP in chickpea production in Isfahan province. Based on Nemecek et al. (2011), emissions of nitrogen dioxide and carbon dioxide from chemical fertilizers and diesel fuel were identified as major contributors to global warming potential.

**Fig. 3:**
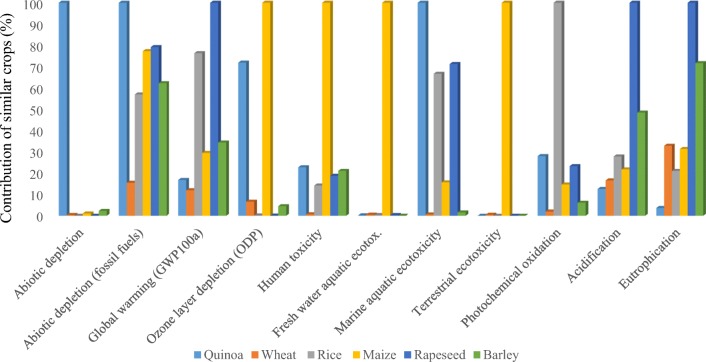
Comparison of quinoa with several similar crops

OLD increases the amount of harmful solar radiation by chlorine, bromine, and chlorofluorocarbons chemicals. This phenomenon causes some serious consequences on the environment and human such as skin cancer, molecular damage to materials, and damage to plants and animals due to increased UV radiation. The impact of greenhouse gases is quantified in OLD impact category (Bare 2011). In the present study, this impact was accounted as 19 mg CFC11_eq._ per defined FU. Figure 3 shows that pesticides with the contribution of 94% to OLD were identified as environmental hotspots. The amount of OLD in canola production was obtained 13 times higher than value in this study, and pesticides are the most effective factor for this impact (Mousavi-Avval et al. 2017). Using physical and biological pest control methods, management of time and pesticide amount and proper operational adjustment of the sprayer will be effective in reducing these pollutants.

The HTP impact indicates the damage potential caused by chemicals emission to the environment. As can be seen in Table 4, HTP was obtained as 280 kg 1,4-DB_eq._ per one tonne of quinoa grain production. The highest contribution to this impact is related to diesel fuel input. The use of reduced tillage and no-tillage cultivation operations in quinoa farms can reduce diesel fuel consumption. Nikkhah et al. (2016) reported that high on-farm emission rates for agricultural productions in Iran could be attributed mainly to diesel fuel combustion and nitrogen fertilizer application. In the present study, phosphorus fertilizers had the highest contribution (65%) on TE impact. Based on Sahle and Potting (2013), the most effective input to the TE impact in Ethiopian roses production was fertilizer with the contribution of 75.5% to the total impact. In the present study, Potassium fertilizers were identified as the only input that had a positive effect on the environmental impacts per one tonne of quinoa production with the most positive effect on TE impact. The PhO impact is mainly caused by the formation of ozone in the lower layers of the atmosphere which is abnormal (Bare et al. 2003). As can be seen in Table 4, the Pho impact was estimated as 161 g C_2_H_4eq._ per defined FU. Nitrogen fertilizer was identified as hotspot in this impact which was followed by diesel fuel. Emissions of NH_3_, NO_2_, SO_2_, and NO_x_ into the air have a significant effect on ACP impact. Based on the results ACP was calculated as 3.2 kg S_2_O_eq._ per production of one tonne quinoa. In the current study, the major contributor to ACP was nitrogen fertilizer application with the share of about 80%. In the rapeseed study by Choobin et al. (2016), phosphate fertilizer accounted for 30% of the total contribution to ACP. The use of crop residue management and proper selection of the right method, the right timing, and the right rate for fertilizer application, precise and desired placement of nutrient materials, use of manure, and other types of bio-fertilizer can reduce the consumption of chemical fertilizer.

Increasing the nitrate and phosphate concentration in water causes unusual algae growth, which reduces the oxygen of water and degrades aquatic ecosystems. This phenomenon is quantified by EU impact. During the production of one tonne of quinoa, EU impact was obtained as 495 g PO_4_^3−^_eq._. Phosphorus has been introduced as a major contributor to the EU in most European ecosystems (Charles et al. 2006).

Comparison of the environmental impacts of quinoa production with wheat, barley, maize, rice, and rapeseed is presented in Fig. 3. The results show that the environmental impacts of quinoa are similar to those of agricultural crops such as wheat, barley, or rice. Quinoa is usually compared with cereals because of its texture and plant origin. However, it has high protein content, as well as all the proteinogenic amino acids, which make it an attractive product to compete with animal protein (Ruiz et al. 2014; Vega-Galvez et al. 2010). However, if the results of environmental impacts are compared with other protein-rich foods (meats, eggs, seafood, etc.), it is observed that quinoa’s environmental impacts are significantly lower than other crops (Cancino-Espinoza et al. 2018). Finally, it must be mentioned that farmers in the Isfahan province have begun to cultivate quinoa, a healthy plant whose glutton-free seeds are rich in protein, dietary fiber, B vitamins, and dietary minerals in amounts greater than in many grains. Growing quinoa has been fully mechanized in the region since its harvest began recently using combine harvesters. Patients with celiac disease who are suffering from gastrointestinal problems can replace wheat with quinoa. By informing people about the benefits of using this plant, its market can be boosted, and it will provide a good incentive to grow this plant, especially in salt lands.

## Conclusion

The present study investigated the energy flow and environmental impacts of quinoa grain production in Isfahan province using the life cycle assessment methodology. The energy pattern indicated the significant effect of chemical fertilizer and diesel fuel inputs on the amount of energy consumed which shows the priority of non-renewable energies. Investigation of energy indices indicated the efficient quinoa production; however, by comparing quinoa energy indices, it was found that the share of energy consumption for quinoa residue production was more than quinoa grain. Inefficient and imprecise use of fertilizers and pesticides has negative impacts on the environment. Some measurements can be recommended for mitigation of environmental impacts such as precise use of agro-chemicals, soil and plant testing for determining fertilizer requirements of soil, and limiting nitrogen cycles by applying suitable crop rotation. Also, the use of modern methods of spraying can reduce the biocides application. The lack of proper machinery and consequently increasing farm traffic as well as faults in applied machinery increase diesel fuel consumption. Development of biofuels production, introduction, and production of multi-fuel engines will greatly help to reduce environmental pollutions. Also, production and introduction of complex and advanced agricultural machinery, software, and monitors and usage of reduced tillage and no-tillage cultivation operations can provide better control over tractor function and reduce operation hours in quinoa cultivation.

## Appendix

**Table 5:** Equations the on-farm emissions related to application of inputs in quinoa production

	Equations (5–16)	(Reference)
1. Chemical fertilizers and manure (kg)	$\mathrm{Relevant}\ \mathrm{fertilizer}\kern0.5em \mathrm{value}\ \left(\frac{\mathrm{kg}}{1\mathrm{ha}\ \mathrm{quinoa}}\right)\times \mathrm{emission}\ \mathrm{factor}\times \left[\frac{\mathrm{kg}\ {\mathrm{N}}_2\mathrm{O}-\mathrm{N}}{\mathrm{kg}\ {\mathrm{N}}_{\mathrm{in}\ \mathrm{fertilizer}}}\right]$	(Nemecek et al. 2014; IPCC 2007)
	$\mathrm{Relevant}\ \mathrm{fertilizer}\kern0.5em \mathrm{value}\ \left(\frac{\mathrm{kg}}{1\mathrm{ha}\ \mathrm{quinoa}}\right)\times \mathrm{emission}\ \mathrm{factor}\times \left[\frac{\mathrm{kg}\ {\mathrm{N}}_2\mathrm{O}-\mathrm{N}}{\mathrm{kg}\ {\mathrm{N}}_{\mathrm{in}\kern0.5em \mathrm{manure}\ \mathrm{applied}}}\right]$	(Nemecek et al. 2014; IPCC 2007)
	$\mathrm{Relevant}\ \mathrm{fertilizer}\kern0.5em \mathrm{value}\ \left(\frac{\mathrm{kg}}{1\mathrm{ha}\ \mathrm{quinoa}}\right)\times \mathrm{emission}\ \mathrm{factor}\times \left[\frac{\mathrm{kg}\ \mathrm{C}{\mathrm{O}}_2-\mathrm{C}}{\mathrm{kg}\ \mathrm{Urea}}\right]$	(Nemecek et al. 2014; IPCC 2007)
	$\mathrm{Relevant}\ \mathrm{fertilizer}\kern0.5em \mathrm{value}\ \left(\frac{\mathrm{kg}}{1\mathrm{ha}\ \mathrm{quinoa}}\right)\times \mathrm{emission}\ \mathrm{factor}\times \left[\frac{\mathrm{kg}\ \mathrm{N}{\mathrm{H}}_3-\mathrm{N}}{\mathrm{kg}\ {\mathrm{N}}_{\mathrm{in}\ \mathrm{fertilizer}\mathrm{s}}}\right]$	(Nemecek et al. 2014; IPCC 2007)
	$\mathrm{Relevant}\ \mathrm{fertilizer}\kern0.5em \mathrm{value}\ \left(\frac{\mathrm{kg}}{1\mathrm{ha}\ \mathrm{quinoa}}\right)\times \mathrm{emission}\ \mathrm{factor}\times \left[\frac{\mathrm{kg}\ \mathrm{N}{\mathrm{H}}_3-\mathrm{N}}{\mathrm{kg}\ {\mathrm{N}}_{\mathrm{in}\kern0.5em \mathrm{manure}\ \mathrm{applied}}}\right]$	(Nemecek et al. 2014; IPCC 2007)
	$\mathrm{Relevant}\ \mathrm{fertilizer}\kern0.5em \mathrm{value}\ \left(\frac{\mathrm{kg}}{1\mathrm{ha}\ \mathrm{quinoa}}\right)\times \mathrm{emission}\ \mathrm{factor}\times \left[\frac{\mathrm{kg}\ \mathrm{N}{\mathrm{O}}_{\mathrm{x}}}{\mathrm{kg}\ {\mathrm{N}}_2{\mathrm{O}}_{\mathrm{from}\ \mathrm{frtilizers}}}\right]$	(Nemecek et al. 2014; IPCC 2007)
	$\mathrm{Relevant}\ \mathrm{fertilizer}\kern0.5em \mathrm{value}\ \left(\frac{\mathrm{kg}}{1\mathrm{ha}\ \mathrm{quinoa}}\right)\times \mathrm{emission}\ \mathrm{factor}\times \left[\frac{\mathrm{kg}\ \mathrm{phosphorous}\ \mathrm{emission}}{\mathrm{kg}\ {\mathrm{phosphorus}}_{\mathrm{in}\ \mathrm{fertilizer}\ \mathrm{and}\ \mathrm{manure}\ \mathrm{applied}}}\right]$	(Nemecek et al. 2014; IPCC 2007)
	$\mathrm{Relevant}\ \mathrm{fertilizer}\kern0.5em \mathrm{value}\ \left(\frac{\mathrm{kg}}{1\mathrm{ha}\ \mathrm{quinoa}}\right)\times \mathrm{emission}\ \mathrm{factor}\times \left[\frac{\mathrm{kg}\ \mathrm{N}{\mathrm{O}}_3^{-}-\mathrm{N}}{\mathrm{kg}\ {\mathrm{N}}_{\mathrm{in}\ \mathrm{fertilizer}\mathrm{s}\ \mathrm{and}\ \mathrm{manure}\ \mathrm{applied}}}\right]$	(Nemecek et al. 2014; IPCC 2007)
2. Diesel fuel	$\mathrm{Fuel}\ \mathrm{energy}\ \left(\frac{\mathrm{MJ}}{1\mathrm{ha}\kern0.5em \mathrm{quinoa}}\right)\times \mathrm{emission}\ \mathrm{factor}$	(Nemecek and Kagi 2007)
3. Residue burning	$\mathrm{Residue}\ \mathrm{burning}\kern0.5em \mathrm{value}\ \left(\frac{\mathrm{kg}}{1\mathrm{ha}\ \mathrm{quinoa}}\right)\times \mathrm{emission}\ \mathrm{factor}$	(Wikström and Adolfsson 2004)
4. Human labor	$\mathrm{Human}\ \mathrm{labor}\ \mathrm{value}\ \left(\frac{\mathrm{man}-\mathrm{h}}{1\mathrm{ha}\ \mathrm{quinoa}}\right)\times \mathrm{emission}\ \mathrm{factor}$	(Pre-Consultants 2014)

## Abbreviations

ACP: Acidification potential
AD: Abiotic depletion
DE: Direct energy
EP: Energy productivity
EU: Eutrophication
ER: Energy ratio
FAD: Fossil fuels abiotic depletion
FAE: Fresh water aquatic ecotoxicity
FU: Functional unit
GWP: Global warming potential
HTP: Human toxicity potential
IDE: Indirect energy
LCA: Life cycle assessment
LCI: Life cycle inventory
LCIA: Life cycle impact assessment
MAE: Marine aquatic ecotoxicity
NEG: Net energy gain
NRE: Non-renewable energy
OLD: Ozone layer depletion
PhO: Photochemical oxidation
RE: Renewable energy
SE: Specific energy
